# Inhibition of α-glucosidase, α-amylase, and aldose reductase by potato polyphenolic compounds

**DOI:** 10.1371/journal.pone.0191025

**Published:** 2018-01-25

**Authors:** Diganta Kalita, David G. Holm, Daniel V. LaBarbera, J. Mark Petrash, Sastry S. Jayanty

**Affiliations:** 1 San Luis Valley Research Center, Department of Horticulture and Landscape Architecture, Colorado State University, Center, United States of America; 2 Department of Pharmaceutical Sciences, Skaggs School of Pharmacy and Pharmaceutical Sciences, University of Colorado Anschutz Medical Campus, Aurora, United States of America; 3 Department of Ophthalmology, University of Colorado Anschutz Medical Campus, Aurora, United States of America; Agriculture and Agri-Food Canada, CANADA

## Abstract

Diabetes mellitus is a chronic disease that is becoming a serious global health problem. Diabetes has been considered to be one of the major risks of cataract and retinopathy. Synthetic and natural product inhibitors of carbohydrate degrading enzymes are able to reduce type 2 diabetes and its complications. For a long time, potatoes have been portrayed as unhealthy for diabetic patients by some nutritionist due to their high starch content. However, purple and red potato cultivars have received considerable attention from consumers because they have high levels of polyphenolic compounds that have potent antioxidant activities. In this study, we screened the total phenolics (TP) and total anthocyanins (TA) and analyzed the phenolic and anthocyanin compounds in selected potato cultivars and advanced selections with distinct flesh colors (purple, red, yellow and white). Purple and red potato cultivars had higher levels of TP and TA than tubers with other flesh colors. Chlorogenic acid is the predominant phenolic acid, and major anthocyanin is composed of the derivatives of petunidin, peonidin, malvidin and pelargonidin. We tested the potential inhibitory effect of potato extracts on the activities of α-amylase and α-glucosidase, which were targeted to develop antidiabetic therapeutic agents. We also measured inhibitory effect of potato extracts on aldose reductase (AR) which is a key enzyme that has been a major drug target for the development of therapies to treat diabetic complications. Purple flesh tubers extract showed the most effective inhibition of α-amylase, α-glucosidase, and aldose reductase with IC_50_ values 25, 42, and 32 μg/ml, respectively. Kinetic studies showed that anthocyanins are noncompetitive inhibitors of these enzymes, whereas phenolic acids behaved as mixed inhibitors for α-amylase and α-glucosidase and noncompetitive inhibitors for AR. This study supports the development of a positive and healthful image of potatoes, which is an important issue for consumers.

## Introduction

Diabetes mellitus (DM) is a chronic disease and is characterized by abnormal glucose tolerance and insulin resistance [1]. DM is associated with complications, such as metabolic syndrome, heart disease, renal function recession, and blindness. Post prandial hyperglycemia is a major risk factor in the development of type II diabetes [2]. One of the most effective methods to prevent diabetes and hyperglycemia is to control the glucose level in blood [3]. Sugars in blood originates from the hydrolysis of carbohydrates and is catalyzed by digestive enzymes, such as α-glucosidase and α- amylase. α-glucosidase is an intestinal cell membrane enzyme whose function is to hydrolyze polysaccharides. Similarly, α- amylase is an enzyme that is secreted by the pancreas and salivary glands that can hydrolyze starches and oligosaccharide into simple sugars. Inhibition of these enzymes can retard carbohydrate digestion, thus causing a reduction in the rate of glucose absorption into the blood. Therefore, inhibition of these enzyme activities in digestive organs is considered to be a therapeutic approach for managing diabetes [4–6]. Aldose reductase is a key enzyme in the polyol pathway. It catalyzes the reduction glucose to sorbitol and provides a common link in the onset of diabetic complications in various parts of the human body. Intracellular accumulation of sorbitol leads to the local hyperosmotic conditions that are responsible for the development of diabetic complications such as cataract, retinopathy, neuropathy, and nephropathy [7]. Therefore, aldose reductase has been an attractive drug target in the clinical management of these diabetic complications [8–10].

Some synthetic inhibitors of these enzymes, such as acarbose and voglibose, have been developed [11]. However, some side effects are seen with these inhibitors, such as flatulence and digestive and liver function disorders. Therefore, inhibitors that have no side effects and come from natural sources are preferred. Many studies have investigated the antidiabetic activities of these phytochemicals in vitro and in vivo [4–10]. Several research efforts have been reported for effective α-amylase and α-glucosidase and aldose reductase inhibition from natural sources to develop a physiological functional food or lead compounds for use in antidiabetic medications [4–8]. Among them, polyphenolic compounds are secondary plant metabolites and constitute the largest group of health-promoting phytochemicals. The compounds that are responsible for the inhibition of α-amylase, α-glucosidase, and aldose reductase include phenolic acid, flavonoids, flavonol and anthocyanins [4–10].

Diets rich in fruits and vegetables are associated with a lower risk of chronic diseases since fruits and vegetables are a good source of polyphenols. Potatoes are one of the major food crops, after rice, wheat, and maize. It has a favorable overall nutrient-to-price ratio compared with many other fruits and vegetables and are an affordable source of nutrition worldwide. Historically, potato plant breeders have focused on traits related to external quality, yield, durability and visual appeal, but rarely on nutritional quality. Developing new potato cultivars with higher levels of nutritional value and bioactive compounds is considered to be a realistic approach to increasing dietary nutritional and antioxidant intake. Breeders and geneticist worldwide are working to enhance the phytonutrient content of potatoes [12–14]. As a result, new potato cultivars with distinctive flesh and skin colors are being developed. Screening for genetic divergence, in terms of health and bioactive compounds among the wild relatives, is a useful tool for plant breeder for selecting an efficient choice of parents for breeding programs. Potato tubers with red and purple flesh are known to have high phenolic contents, significant antioxidant properties [15, 16] and anticancer activity [17]. Due to these potential health attributes, specialty/colored flesh potato consumption is continuously increasing over recent years. Still, potatoes are often maligned in nutrition circles because of their suspected cause to obesity and diabetics. There are no detailed reports on the potential role of potatoes in preventing diabetes and its complications. The study was aimed to investigate the potential role of potato polyphenolic compounds in the inhibition of α-amylase, α-glucosidase and aldose reductase activities for the implication of antidiabetic properties.

## Materials and methods

**Fig 1:**
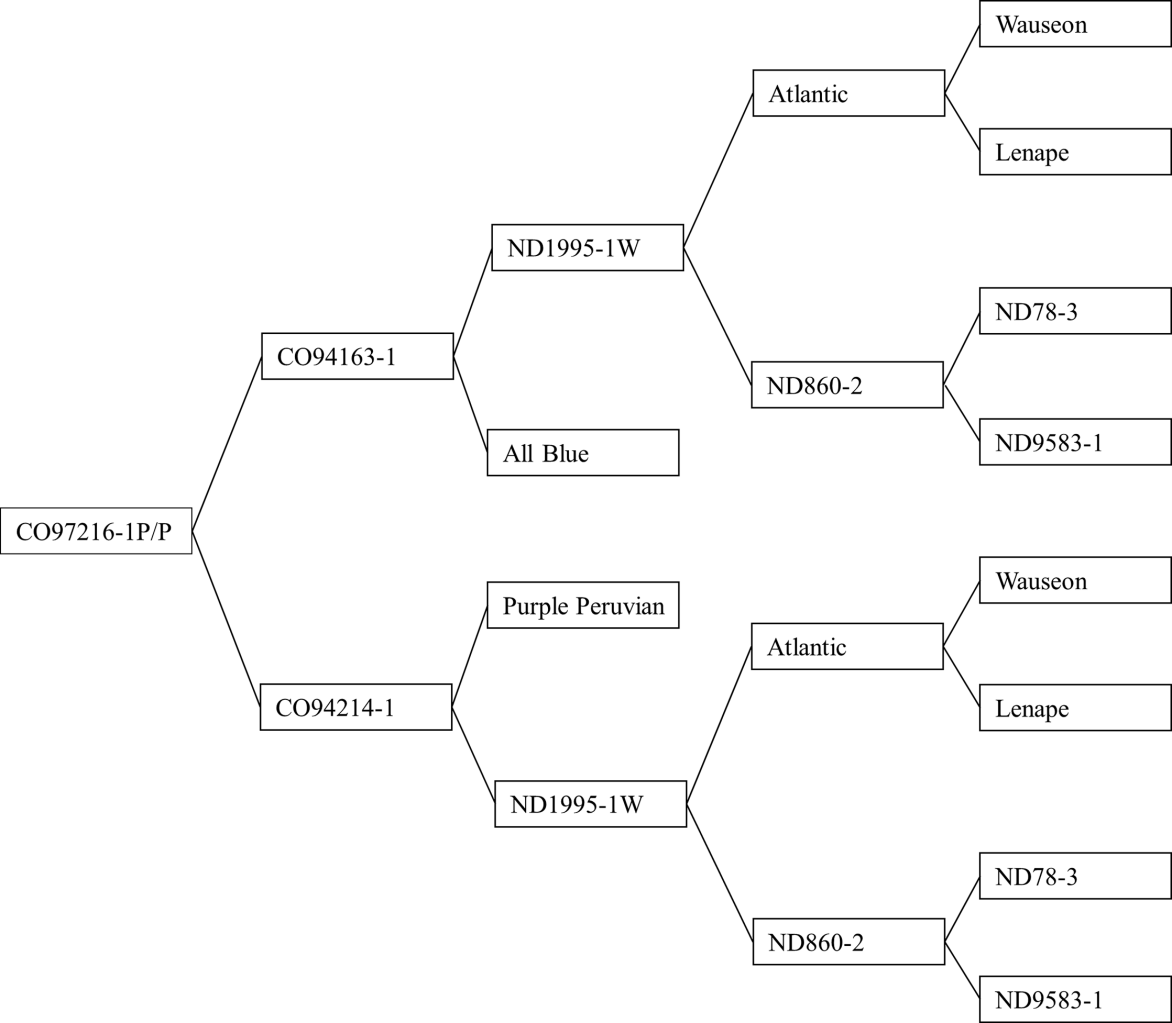
Pedigree chart for the potato cultivar CO97216-1P/P.

Purebred potato tubers Purple Majesty, CO97216-1P/P, CO97222-1R/R, CO97226-2R/R, Masquerade, Yukon Gold, Rio Grande Russet, and Russet Nugget with distinctive flesh colors used in this study are the product of Colorado potato breeding program at San Luis Valley Research Center (SLVRC), Colorado State University. They were resulted from the crosses made between specific male and female (Table 1) produced from earlier crosses. Fig 1 represents the pedigree for CO97216-1P/P. Twenty potato tubers of each clone were stored in a cooler at 10°C with above 90% humidity after harvesting from the field. All chemicals were purchased from Sigma Aldrich, St Louis, USA. Recombinant human aldose reductase was produced as previously described by Tarle et al. [18].

**Table 1:** Selected potato cultivars and physical appearance.

clones	skin color	flesh color	parents (Female X Male)
CO97222-1R/R	Red	Red	CO94170-1 X Mountain Rose
CO97226-2R/R	Red	Red	Mountain Rose X CO94214-1
Purple Majesty	Red	Purple	ND2008-2 X All Blue
CO97216-1P/P	Purple	Purple	CO94163-1 X CO94214-1
Yukon Gold	White	Yellow	Norgleam X W5279-4
Masquerade	White and purple	Yellow	Inka Gold X A91846-5R
Rio Grande Russet	White	White	Butte X A8469-5

### Extraction of polyphenolic compounds

Phenolics and anthocyanins were extracted from potato tubers stored as mentioned above. Two-hundred grams of freeze-dried material was weighed into a 500-ml conical flask, and 250 ml of aqueous 80% methanol was added. The mixture was stirred overnight on a magnetic stirrer plate at 25°C. The mixtures were filtered through Whatman 100 filter paper. The residues were re-extracted under the same conditions. The combined filtrates were evaporated using a rotary evaporator until all of the methanol was evaporated. The final residue was lyophilized and kept at -80°C prior to analysis.

### Analysis of total phenolics

A 20-μl aliquot of methanolic extract was mixed with 50 μl of distilled water in a 96-well flat-bottom assay plate. 50 μl of a commercial FCR solution (MP Biomedical, Solon, OH) was added and mixed well for 1 min in a plate reader (Power Wave XS2, BioTek Instruments, Winooski, VT). After 5 min, 80 μl of a 175 gml^-1^ sodium carbonate solution was added and immediately mixed with a pipette. The plates were incubated at 25°C in the dark with agitation at 150 rpm on an orbital shaker for two hours. The absorbances of the content were measured at 760 nm. The gallic acid in methanol was used as the standard, and the total phenolic values were quantified as micrograms of gallic acid equivalent per gram of dry weight material.

### Analysis of total anthocyanin

The total monomeric anthocyanin was determined by the pH differential method. A 10-μl aliquot of the methanol extract of potato tubers was added separately to 290 μl of potassium chloride (pH 1.0) and sodium acetate (pH 4.5) buffers. The absorbance was measured at 515 nm and 700 nm for both sets of pH 1.0 and 4.5 solutions using water as a blank. The total anthocyanin content was calculated using the following equation:

$$\begin{array}{l} \mathrm{Total}\;\mathrm{monomeric}\;\mathrm{anthocyanin}=(A\;x\;\mathrm{MW}\;x\;\mathrm{DF}\;x\;1000)/\in x\;l\\ \mathrm{Where}\;A=(A_{515}‑A_{700})\;\mathrm{pH}\;1.00‑(A_{515}‑A_{700})\;\mathrm{pH}\;4.5 \end{array}$$

where 449.2 and 26,900 are the molecular weight and molar absorptivity of cyanidine-3-glucoside (C3G), respectively, which was used as the standard; DF is the dilution factor, and l is the path length. The total μg/g dry weight of potato tuber anthocyanin was reported.

### Analysis of chlorogenic acids

Three isomers of chlorogenic acid viz 5-caffeoylquinic acid (5CQA), 4-caffeoylquinic acid (4CQA), and 3-caffeoylquinc acid (3CQA) were quantified using Water 2695 HPLC spectrophotometer with a quaternary pump with the detector photodiode array and Nova Pak^®^ C18 column. The mobile phase consisted 1% phosphoric acid (A) and acetonitrile (B) with a gradient 0–1 min 90% A and 10% B,1–20 min 30%A and 70%B and absorbance was monitored at 210–400 nm. The injected sample volume was 10 μl. 5CQA, 4CQA, and 3 CQA were quantified using the calibration curve for standard 5CQA, 4CQA and 3CQA.

### Fractionation

**Fig 2:**
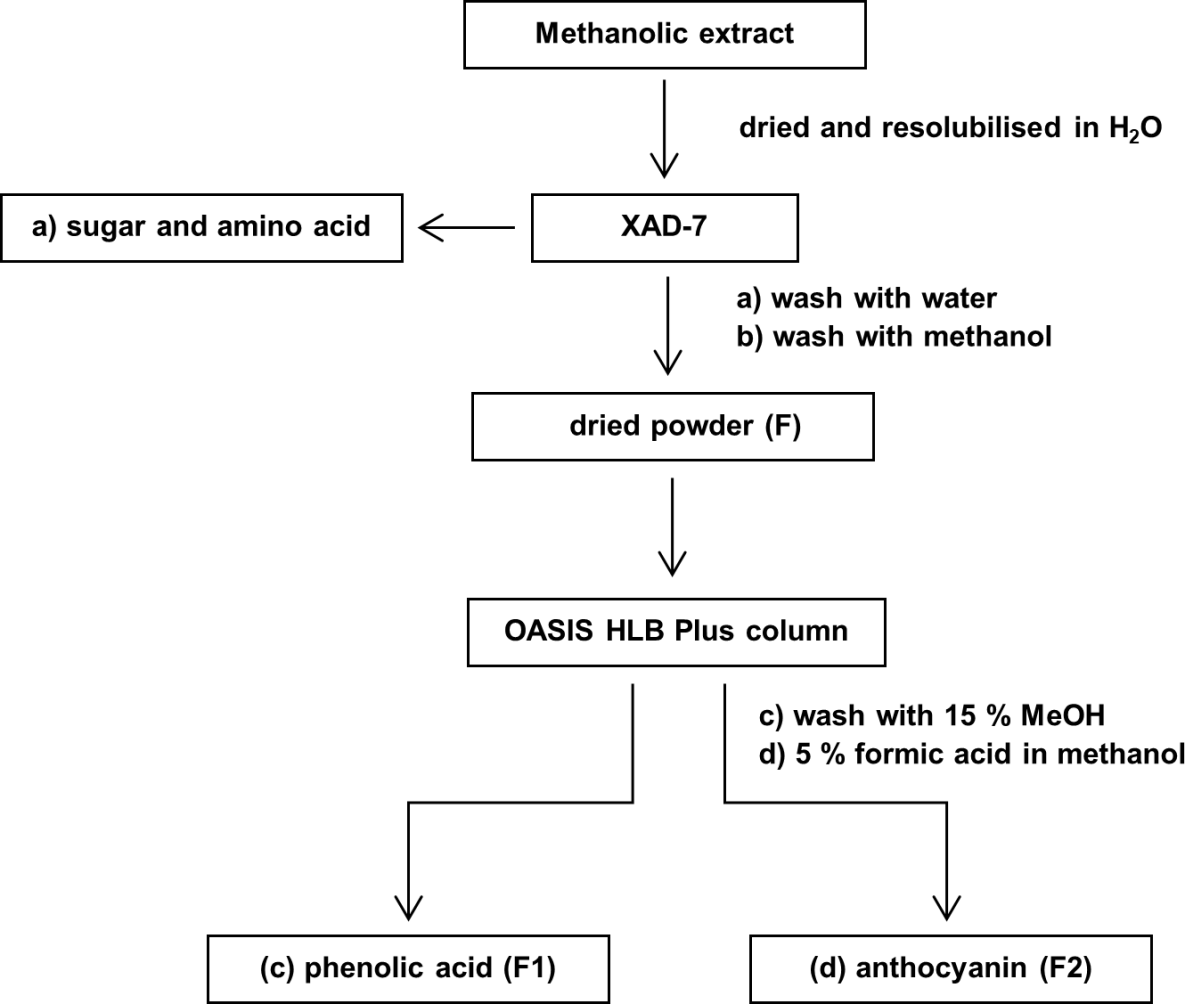
A flow diagram representing the purification and separation of methanolic extract of potato tubers.

Five grams of the dried methanolic extract were dissolved in minimum water and then loaded onto an Amberlite XAD-7 column. The adsorbent was washed with water to remove sugars organic acids and salt. Phenolic compounds and anthocyanins retained in the adsorbent and eluted with methanol. The eluent was concentrated to dryness with a Büchi rotary evaporator connected to a high vacuum at 40°C. After evaporation, the remaining portion was placed in a freeze drier to remove the remaining water. Further, the XAD-7 extract was re-dissolved in a minimum volume of water and applied into OASIS HLB Plus cartridge. Two fractions: a) phenolic acid and b) anthocyanins were eluted with methanol and formic acid-methanol. Fig 2 represents the separation and isolation of these two fractions.

### LC-MS chromatogram

One milligram of each fraction (1 mg) were suspended in 1 ml of methanol-water (7:3). Five microliters of extract were injected into a Waters Acquity UPLC system and separated using a Waters Acquity UPLC HSS T3 column (1.8 μM, 1.0 x 100 mm) with a gradient from solvent A (water, 0.1% formic acid) to solvent B (Acetonitrile, 0.1% formic acid). Injections were made in 100% A, held at 100% A for 1 min, ramped to 98% B over 12 minutes, held at 98% B for 3 minutes, and then returned to the starting conditions over 0.05 minutes and allowed to re-equilibrate for 3.95 minutes, with a 200 μL/min constant flow rate. The column and samples were held at 50°C and 5°C, respectively. The column eluent was infused into a Waters Xevo G2 Q-TOF-MS with an electrospray source in either positive or negative ionization mode, scanning 50–1200 m/z at 0.2 seconds per scan, alternating between MS (6 V collision energy) and data-dependent MS/MS mode (15–30 V ramp). For positive ionization mode, the cone voltage and capillary voltages were set to 30 V and 2.2 kV, respectively. For negative ionization mode, the cone voltage and capillary voltages were set to 20 V and 2.0 kV, respectively. For both ionization modes, the source temp was 150°C and the nitrogen desolvation temperature was 350°C with a flow rate of 800 L/hr. Calibration was performed using sodium formate with a 1 ppm mass accuracy.

### Inhibition of α-glucosidase, α-amylase and aldose reductase activities

α-glucosidase activity was assayed spectrophotometrically using PNPG as a substrate. The continuous production of p-nitrophenol was determined by measuring the absorbances at 405 nm in a reaction mixture containing 1U/ml glucosidase and 1mM PNPG incubated in pH 6.5 phosphate buffer. The initial reaction rate of PNPG hydrolysis in the absence of inhibitors, Vo was determined and was used as a control. The effect of a XAD-7 extract of each potato cultivar was assessed by adding different concentrations (10–200 μg/ml) in the glucosidase assay. The IC_50_ values were graphically determined as the half-maximal inhibitory concentration of the inhibitor species giving 50% inhibition. All assays were performed in triplicate.

α-amylase activity measurements were performed at 37°C using 2-chloro-4nitrophenyl α-D-maltotrioside (CNPG3) as a substrate. A total of 100 μl of α-amylase solution (1 μg/ml in phosphate buffer) was added to a reaction mixture containing 2.0 mM CNPG3, 200 mM sodium chloride, 5.0 mM calcium chloride, and 50 mM phosphate buffer at pH 6.5. The progress of the reaction was monitored by the absorbance at 405 nm for the production of chloro-nitrophenol in the presence of different concentrations of (10–200 μg/ml) sugar and organic acid free methanolic extract.

Aldose reductase activity was assayed using glyceraldehyde as a substrate and NADPH as a cofactor as described previously [19]. Inhibition assays contained a range of XAD-7 concentrations, keeping the final methanol concentration equivalent among reaction mixtures. Reaction rates were measured by monitoring the absorbance at 340 nm, and were determined in triplicate for each concentration of XAD-7. Reaction rates were compared to control assays not containing methanolic extract to determine inhibition.

### Kinetics of inhibition

The mode of enzyme inhibition by the potato extract compounds was determined by using the Michaelis-Menten and Lineweaver- Burk equations. PNP glycosides at concentrations ranging from 1 to 5 mM were used as substrates for α- glucosidase and α-amylase respectively. The enzyme activities were determined in the absence or presence of different concentrations of compound fractions. The concentrations of phenolic and anthocyanin compounds used for the inhibitory kinetics of amylase were 10, 20, 30 and 50 μg/ml for both enzymes. The kinetic parameters *V*_*max*_ and *K*_*m*_ were determined using the Lineweaver- Burk plot (LB plot) following the Michaelis-Menten equation.

$$\frac{1}{V}=\left\{\frac{Km}{V\max\left[S\right]}+\frac{1}{V\max}\right\}\;\frac{1}{V}=\left\{\frac{Km}{V\max\left[S\right]}+\frac{1}{Vm\mathrm{ax}}\right\}$$

Where *V*_*max*_ is the maximal velocity and K_m_ is the Michalis constant, and its value is equivalent to the substrate concentration at which the velocity is equal to one half of the velocity. *V*_*max*_ and *K*_*m*_ were obtained from the intercept and slope, respectively. The expression of the velocity of the reaction in the present case is given by

$$v=\left\{\frac{V\max X\left[S\right]}{Km\left(\frac{1+\left[I\right]}{Ki}\right)+\left[S\right]\left(\frac{1+\left[I\right]}{Kii}\right)}\right\}$$

Where *V* is the velocity, [S] is the substrate concentration of PNPG and CNP-G3, [I] is the inhibitor concentration, *K*_*i* is_ the inhibitory constant for the competitive part, and *K*_*ii*_ is the inhibitory constant for the noncompetitive part. The enzyme inhibitor and enzyme substrate inhibitor constants were calculated from secondary plots of the initial rate data by linear regression analysis. Plotting of slopes obtained from the LB plots vs inhibitor concentrations gave the K_i_ values, and intercepts vs inhibitor concentrations gave the K_ii_ values from the x- intercepts.

### Statistical analysis

All experiments were carried out in triplicate, and statistical analyses were performed by one-way analysis of variance (ANOVA) at a p ≤ 0.05 significance level using XLSTAT 2015 (Addinsoft USA, New York, NY). The error bars in the figures are based on the calculated Fishers LSD at α = 0.05 using the standard error for the LS means and were approximated at a T-value of 2.

## Results and discussion

### Total phenolic (TP) and total anthocyanin (TA) contents

**Fig 3:**
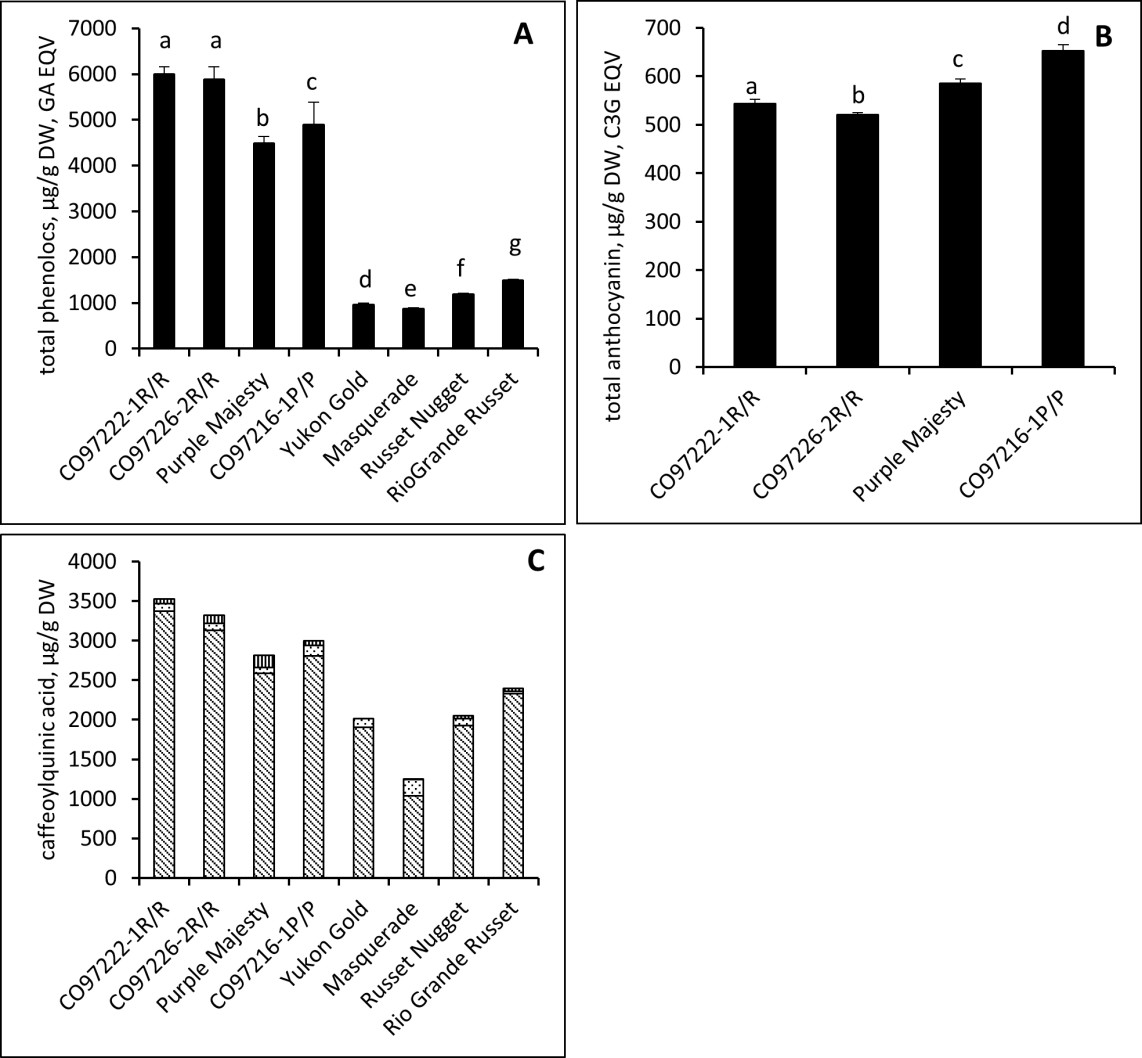
Amount of total phenolic (TP) and total anthocyanins (TA) in potato cultivars. Error bars represent the calculated LSD for the least square means given at α = 0.05. Significant differences are denoted by different letters, same or shared letters indicate that they are not significant to each other.

Selected potato tubers were evaluated for phenolics and anthocyanin as previous earlier reports had suggested that phenolics and anthocyanins are some of the major compound groups that constitute potato tubers and they impart extra health benefits. These groups of compounds were extracted with methanol as solvents. We measured TP and TA in these extracts and the results are shown in Fig 3 (S1 Table). The TP content was in the order CO97222-1R/R > CO97226-2R/R > CO97216-1P/P > Purple Majesty > Rio Grande Russet > Russet Nugget > Yukon Gold > Masquerade, with all of the values being significantly different at p < 0.05 except CO97222-1R/R and CO97226-2R/R. In general, red and purple potato tubers had higher phenolics than white and yellow potato tubers. Similar observations were reported that potato tubers with red flesh had a higher phenolic content than tubers with purple flesh [15]. Previous reports also indicated that red and purple-fleshed potato tubers had total phenolic contents comparable to phenolic-rich fruits and vegetables, such as berries and grapes [20, 21]. As chlorogenic acid constitute approximately 80% of phenolic acid and we have measured the levels of three isomeric forms of chlorogenic acid viz 5-caffeoylquinic acid (5CQA), 4-caffeoylquinic acid (4CQA), and 3-caffeoylquinic acid (3CQA) in the selected potato cultivars and results are shown in Fig 3C. 5-caffeoylquinc acid levels were comparatively higher than the other two isomers. The order of chlorogenic acids in the potato cultivars are CO97222-1R/R > CO97226-2R/R > CO97216-1P/P > Purple Majesty > Rio Grande Russet > Russet Nugget > Yukon Gold > Masquerade.

Anthocyanins are reported to have significant antioxidant activity and antidiabetic properties and highly abundant in fruits and vegetables [22]. The presence of these compounds is the main contributor to the red and purple color of the potato tubers. The total anthocyanin contents were found to be higher in purple potato tubers than red potato tubers in the order CO97216-1P/P > Purple Majesty > CO97222-1R/R > CO97226-2R/R (S1 Table). All of the values were significantly different at p < 0.05. In the literature, very few reports are available on the anthocyanin content potato tubers with colored flesh [13]. Recently, we demonstrated that purple flesh potato tubers have an anthocyanin content comparable to that of blueberries and pomegranate [21].

### Fractionation and characterization

**Fig 4:**
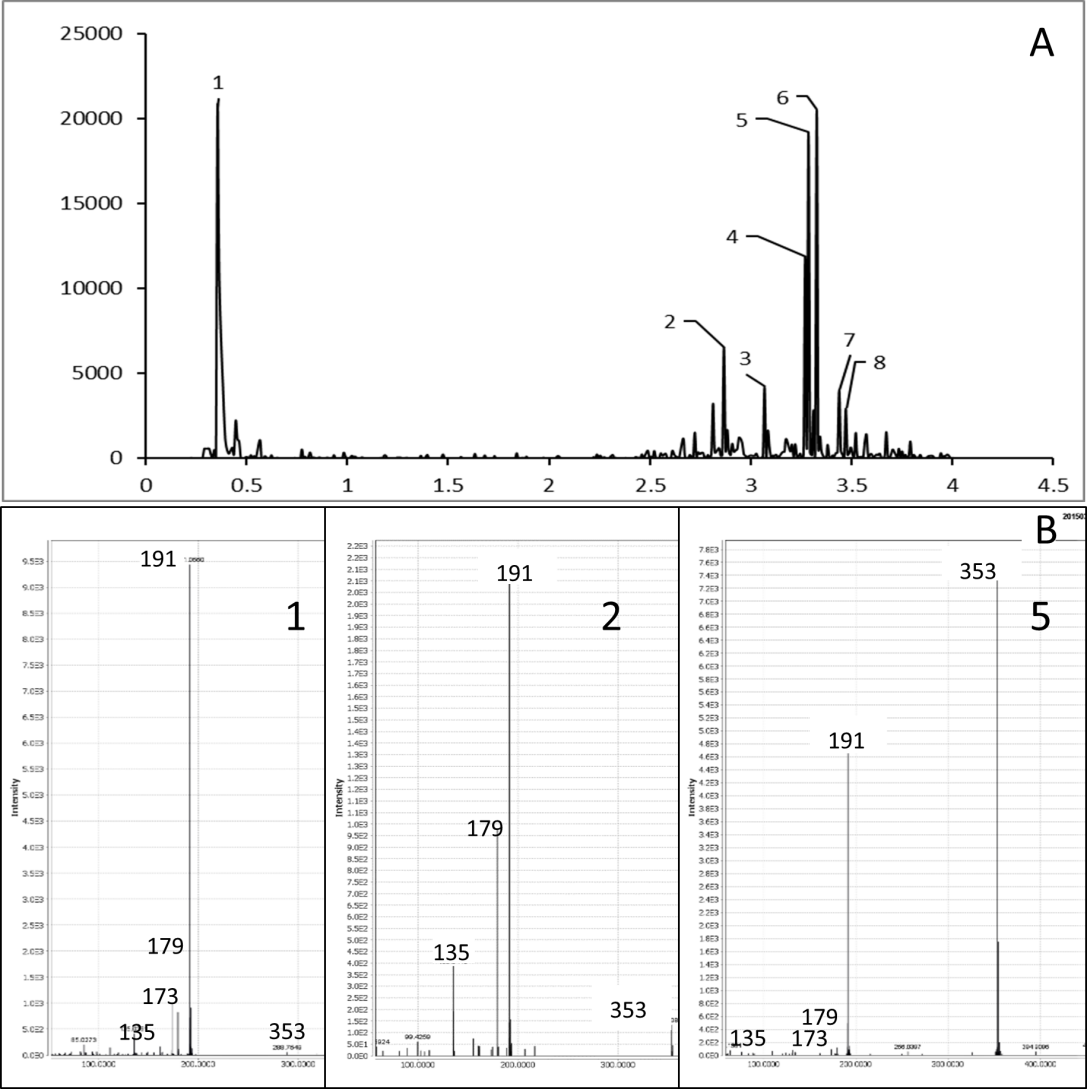
Total ion chromatogram (TIC) of F1fraction from CO97216-1P/P tubers in ESI—ve mode (A). MS/MS spectra of peaks 1,2, and 5 (B).

**Fig 5:**
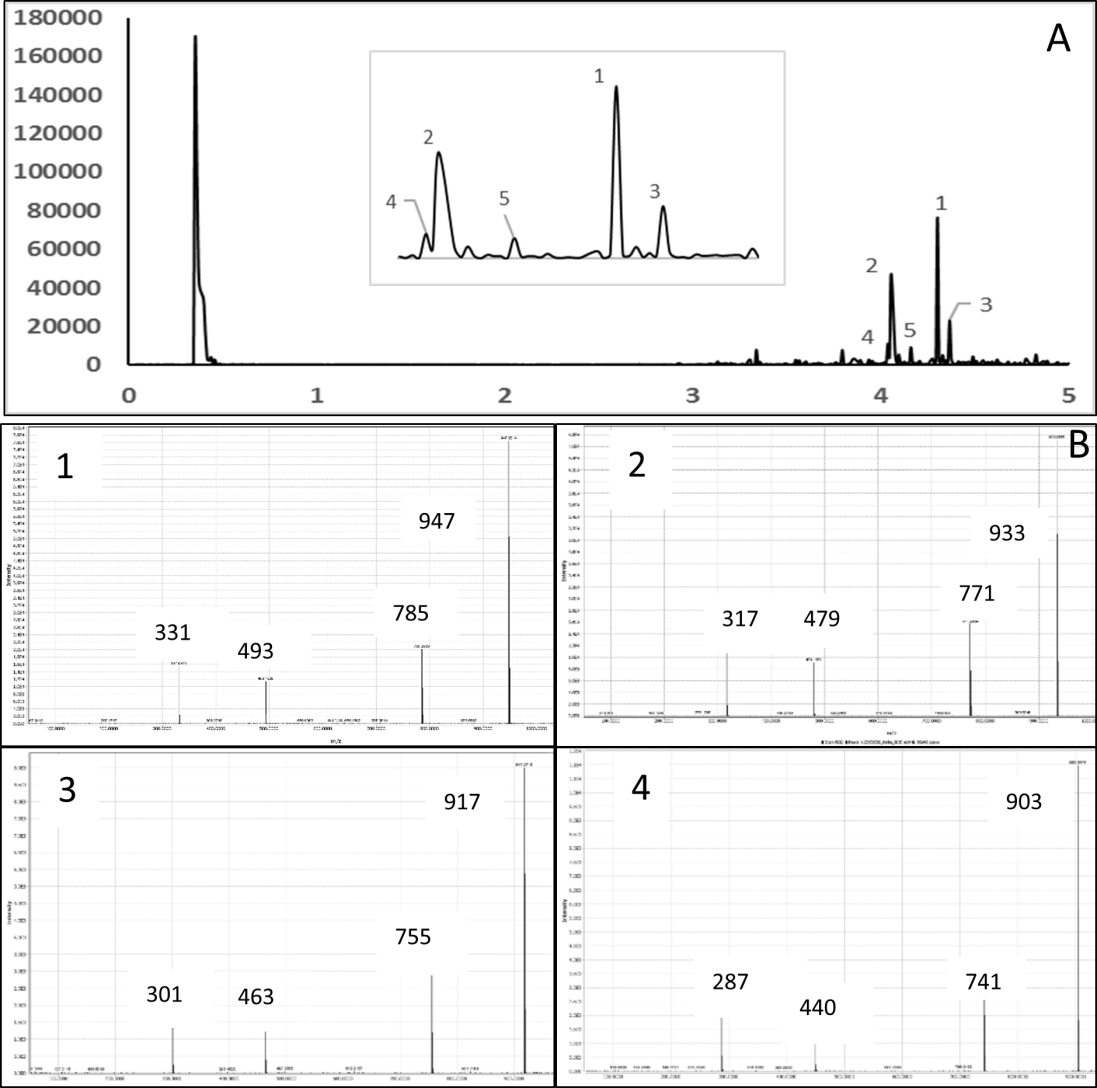
Total ion chromatogram (TIC) of F2 fraction from CO97216-1P/P tubers in ESI + ve mode (A). Inset: enlarged chromatogram of the selected area.MS/MS spectra of each peak (B).

Our previous and other studies reported that purple and red flesh potato tubers have a high level of polyphenolic compounds [12, 13, 15, 21]. However, most of these studies were characterization and analysis methanolic crude extract and their potential antioxidant activities. In this study, we were interested in separating and isolating phenolic acids and anthocyanins. As shown in the flow diagram in Fig 2, sugars, amino acids and other water soluble nonphenolic compounds were removed using an Amberlite XAD-7 column. The compounds remained in the adsorbent were eluted with methanol and then further purified by an Oasis HLB plus column. Eluting the column with 15% MeOH and 5% formic acid in methanol separated into phenolic acids (F1) and anthocyanins (F2), respectively. Moreover, a group of compounds present in F1 and F2 were characterized by LCMS in ESI–ve and +ve mode, respectively and results are shown in the Table 2 taking CO97216-1P/P and CO97222-1R/R as representative potato cultivars. The TIC of F1 and F2 and selective spectra from CO97216-1P/P are shown in Figs 4 and 5 respectively.

**Table 2:** Phenolic and anthocyanin compounds present in purple and red potato tubers.

Potato cultivar	phenolic acid and anthocyanins	Molecular ion, (m/z)	MS/MS(m/z)	% composition
CO97216-1P/P	chlorogenic acid	353	191, 179, 173, 135	-
	petunidin-3-coumaroylrutinoside-5-glucoside	933	479,771, 317	28.24
	Peonidin-3-coumaroyllrutinoside-5-glucoside	917	755, 463, 301	13.82
	malvidin-3-coumaroylrutinoside-5-glucoside	947	785, 493, 331	45.91
	cyanidin-3-coumaroylrutinoside-5-glucoside	903	741, 433, 287	6.61
CO97222-1R/R	Chlorogenic acid	353	191, 179, 173, 135	-
	Pelarogonidin-3-coumaroylrutinoside-5-glucoside	887	725, 433, 271	88.67
	Pelarogonidin-3-caffeoylrutinoside-5-glucoside	903	741, 433, 271	6.82
	Pelarogondin-3-feruloylrutinoside-5-glucoside	917	755, 433, 271	4.5

**Fig 6:**
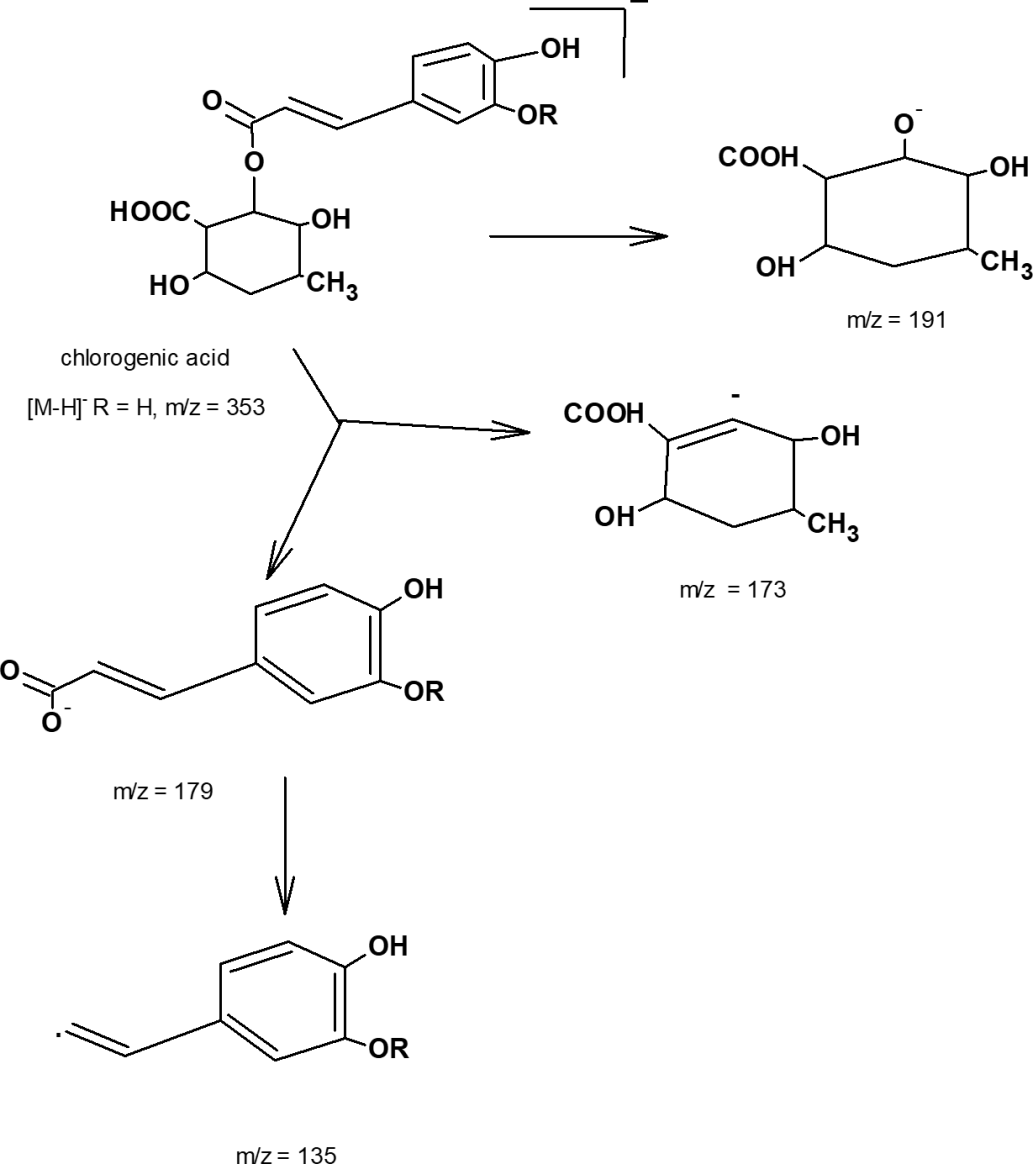
Fragment pattern for chlorogenic acid in MS/MS.

In ESI negative ion mode F1 fraction had major compounds 1–8 and had [M-1]^-^ ion with m/z 353 in accord with the molecular formula for chlorogenic acid C_16_H_18_O_9_ with different retention times (Fig 4A). In MS/MS it gave the fragments with m/z 191, 179 and 135 with varying intensities which are the characteristic fragments of chlorogenic acid isomers. Fig 4B represents the typical spectrum of peaks 1, 2 and 5 from the TIC chromatogram. The relative intensities of the characteristic fragments of each the peak are given in Table 3. Chlorogenic acids are the esterified form of caffeic acid and quinic acid. Fragmentation of chlorogenic acid in negative mode under specific LCMS experimental condition lead to cleavage of intact caffeoyl (m/z 179 and 173) and quinic acid (m/z 191) fragments (Fig 6). 3-caffeoylqunic acid, 4-caffeoylquinic acid, and 5-caffeoylquinic acid are the common isomers of chlorogenic acid and highly abundant in various fruits and vegetables. It has been reported that exposing UV light, chemical and biochemical treatment of tissues and electric field during MS data acquisition leads to isomeric transformation of chlorogenic acid [23]. Therefore, with their cis, trans stereoiosomeric form chlorogenic acid yields various number of isomers. Fang et al identified six different isomers of chlorogenic acid in dried plums based on the nature and intensities of fragmented ions in LC/MS/MS spectra [24]. In a similar study Ncube et al. reported the profiling of 10 isomers of chlorogenic acid in *Nicotiana tabacum* tissues [25]. In our current study major phenolic acid compound present in red and purple fleshed potato tubers were chlorogenic acid and its isomers. Previous studies on the characterization of phenolic acid compounds in potato tubers also reported that chlorogenic acid constituted with its different isomers [13]. In this study, we have identified eight isomers and quantified 3 major isomers of chlorogenic acid in the F1 fraction of CO97216-1P/P.

**Table 3:** Phenolic and anthocyanin compounds present in purple and red potato tubers Relative intensities of characteristic fragment of chlorogenic acid in ESI MS/MS spectra.

	Fragment ion intensities				
Compounds	353	191	179	173	135
1	73	9400	820	1000	41
2	130	2100	980	26	390
3	250	1600	680	120	130
4	51	6400	120	260	110
5	7600	4700	120	88	40
6	7600	5300	180	150	0
7	25	1800	100	270	34
8	34	1400	120	30	38

Analysis of F2 fraction in positive mode by LC-MS revealed the presence of acylated anthocyanins. From the ESI-MS/MS TIC (Fig 5) we found that major anthocyanins present in the purple fleshed tuber CO97216-1P/P were petunidin-3-coumaroylrutinoside-5-glucoside (28.24%), peonidin-3-coumaroylrutinoside-5glucoside (13.82%), malvidin-3-(p-coumarylrutinoside)-5-glucoside (45.91%) and cyanidin-3-coumaroylrutinoside -5 glucoside (6.61%) with M+ values of 933, 917, 947, and 903. These anthocyanins were identified based on their relative nature and intensities of characteristic fragmented ions as given in Table 2. Relative abundancy was calculated based on peak intensities. The MS/MS spectra of each peak are shown in the Fig 5B. Red fleshed cultivar CO97222-1R/R contained mainly pelarogonidin-3-coumaroylrutinoside-5-glucoside (88.67%) with m/z for M+ 887 and fragments 725, 433 and 271. Other anthocyanins found were pelargonidin caffeoylrutinoside-5-glucoside (6.82%) and pelargonidin-3-ferroylrutiniside-5-glucoside (4.5%). Some previous reports described the presence of such group anthocyanin compounds in red and purple potatoes [13, 26–28]. However, the availability and the relative abundancy of these compounds depends on cultivars and agronomic conditions [13, 26–28].

### Effect on α- amylase, α-glucosidase, and aldose reductase activity

#### IC_50_ values for α- amylase, α-glucosidase, and aldose reductase activity

The effect of the XAD-7 methanolic extract on the activity of α- amylase, and α-glucosidase was investigated using PNPG and CNPG-3substrates. The inhibitory effect on aldose reductase was investigated by using glyceraldehyde as a substrate and NADPH as a cofactor. Each extract inhibited the activities of α-amylase, α-glucosidase and aldose reductase in a dose-dependent manner. Acarbose was used as positive control for inhibition of α-glucosidase and α-amylase whereas quercetin was used against aldose reductase. To quantify the inhibitory potential of the extracts, we determined the half-maximal inhibitory concentration (IC_50_) for each fraction that gave rise to 50% suppression of the original enzyme activity. On the basis of IC_50_ values it was found that purple and red flesh potato tubers have significantly more potential for inhibiting these enzyme activities (Table 4 and S1 Table) and with the following order of potency CO97216-1P/P > Purple Majesty > CO97222-1R/R > CO97226-2R/R > Rio Grande Russet > Russet Nugget > Masquerade > Yukon Gold.

**Table 4:** IC_50_ values of the XAD-7methanolic extract for inhibition of and α-glucosidase, α-amylase and aldose reductase. The values represent the three sets of data with standard deviation. Significant differences are denoted by different letters, same or shared letters indicate that they are not significant to each other.

Potato clones	α-amylase (μg/ml)	α-glucosidase (μg/ml)	aldose reductase (μg/ml)
CO97222-1R/R	30.82 ± 1.34^a,c^	45.98 ± 0.53^a^	39.55 ± 0.95^d^
CO97226-2R/R	33.81 ± 0.83^c^	47.74 ± 0.65^c^	46.06 ± 0.64^c^
Purple Majesty	28.99 ± 1.35^a^	45.2 ± 0.60^a^	35.57 ± 0.98^a^
CO97216-1P/P	25.52 ± 0.79^b^	42.42 ± 0.94^b^	32.48 ± 0.78^b^
Yukon Gold	58.48 ± 1.11^e^	62.79 ± 0.39^e^	58.72 ± 0.47^f^
Masquerade	55.50 ± 0.59^d^	58.12 ± 0.68^d^	49.16 ± 0.52^e^
Russet Nugget	41.34 ± 0.89^g^	78.65 ± 0.48^g^	126.31 ± 5.01^h^
Rio Grande Russet	44.86 ± 1.19^f^	75.11 ± 0.21^f^	172.76 ± 3.89^g^
Acarbose	12.86	15.65	-
Quercetin	-	-	13.87

There have been many reports of natural compounds as α-glucosidase inhibitors [4–10]. Secondary plant metabolites such phenols, polyphenols, and anthocyanin attributed potential health benefits through their antidiabetic, anti-carcinogenic, anti-inflammatory properties. We hypothesized that presence of phenolic acids and anthocyanins in purple and red flesh purple potato tubers would have significant inhibitory effect on α-glucosidase, α- amylase, and aldose reductase activities. In support of this hypothesis, we observed that methanolic extract of the selected potato tubers had significant properties with IC_50_ from 25 to 172 μg/ml towards inhibition of the activities of these enzymes. It was observed that the extent of inhibition correlated with the levels of chlorogenic acid and anthocyanins. Purple and red flesh potato cultivars had a higher level of chlorogenic acids than the white and yellow ones, and subsequently, they have higher activity towards the inhibition of these enzyme’s activities. Moreover, purple flesh potato tubers had higher levels of anthocyanin compared to other color flesh potato tubers. The combination of phenolic acid and anthocyanin in the extracts of CO97216-1P/P made the strongest inhibition of α-glucosidase, α- amylase, and aldose reductase activities. A positive correlation between the polyphenolic content of the natural extract and inhibition of glucosidase and amylase activity were also seen in some previous reports [4–6].

#### Mode of inhibition on α- amylase, α-glucosidase, and aldose reductase activity

Most of the previous studies on inhibitory activities of natural compounds on α-amylase, α-glucosidase and aldose reductase were tested with the crude extracts and their IC_50_ values [4–10]. While IC_50_ values showed the potency of a natural compounds, more valuable information were obtained by the kinetics of inhibition by group or individual compounds isolated form natural extract. A number of bioactive compounds such as chlorogenic acid, caffeic acid, kaempferol, luteolin, (+)-catechin/(-)-epicatechin, betaglucogallin have been reported as active inhibitors of α-amylase, α-glucosidase and aldose reductase [4–10]. Inhibitory activities with their mode of the kinetics of inhibition have also been reported for natural product containing a mixture of compounds. For instance the methanolic extract of finger millet showed strong inhibition towards glucosidase and pancreatic amylase with noncompetitive inhibition [29], polyphenol extracts of Maqui (*Aristotelia chilensis*) leaves acted as mixed inhibitors of α-amylase and noncompetitive inhibitors of α-glucosidase [30], Pine bark extract (PBE) non-competitively inhibited human salivary and porcine pancreatic amylases [31], *Gymnema montanum* (GLEt) leaves extract showed competitive inhibition [32] and Muscadine anthocyanin extract inhibited α-glucosidase in competitive nature with K_i_ and IC_50_ as low as 0.5 mg/ml and 1.5 mg/ml [33]. In our study we have demonstrated the mode of inhibition by phenolic acids and anthocyanin compounds isolated from potato extract.

**Fig 7:**
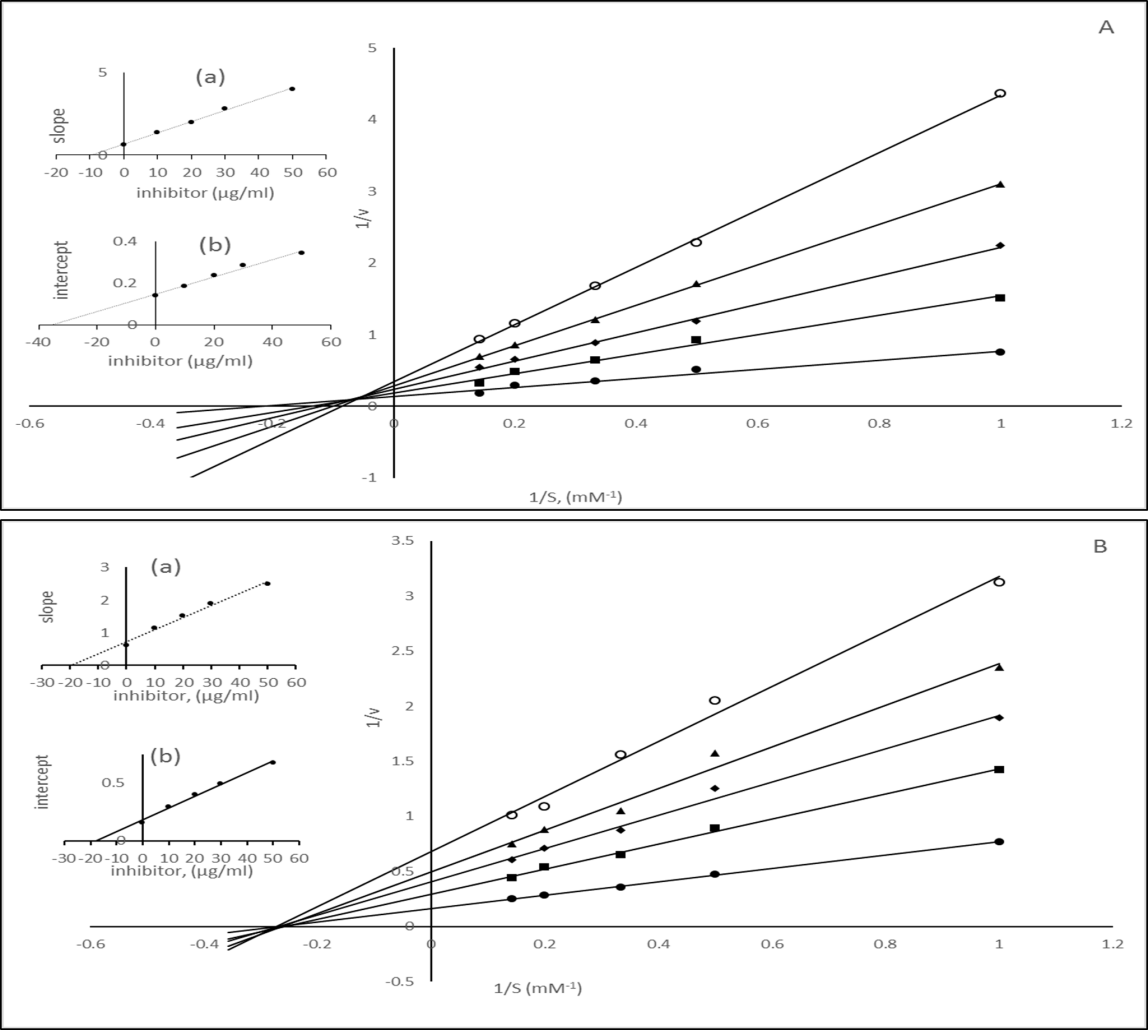
Lineweaver-Burk plot for the activities of α-glucosidase in the presence of various concentration of substrates (1–5 mM) and inhibitors. A) different concentrations of phenolic acids (● 0, ▪ 10, ◊ 20 Δ 30, ○50 μg/ml) B) different concentrations of anthocyanins (● 0, ▪ 10, ◊ 20 Δ 30, ○50 μg/ml).

**Fig 8:**
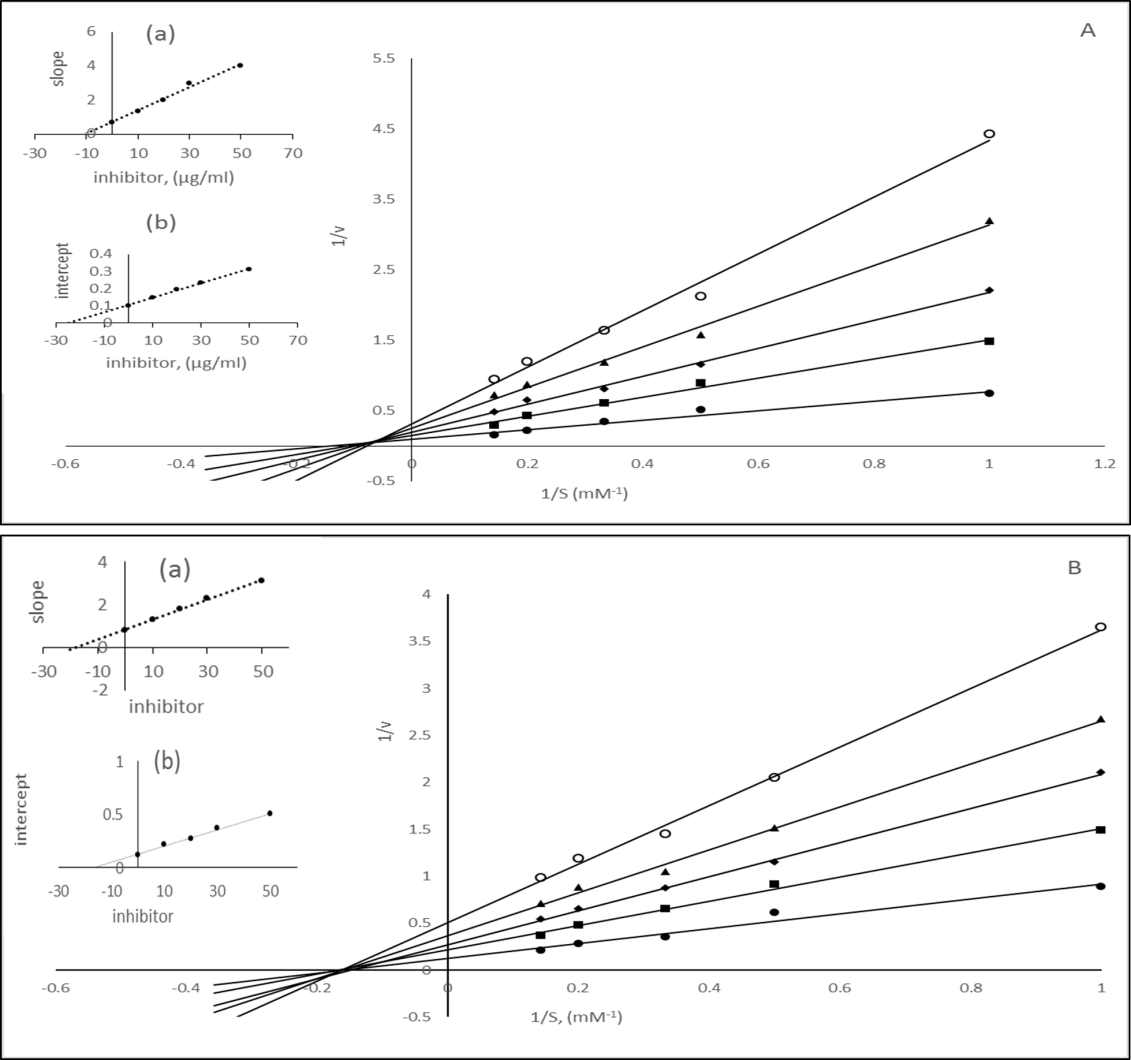
Lineweaver-Burk plot for the activities of α-amylase in the presence of various concentration of substrates (1–5 mM) and inhibitors. A) different concentration of phenolic acids, (● 0, ▪ 10, ◊ 20 Δ 30, ○50 μg/ml) B) different concentration of anthocyanins (● 0, ▪ 10, ◊ 20 Δ 30, ○50 μg/ml).

An inhibitor can interact with an enzyme in various ways. Studies on the kinetics of inhibition are a major tool that enables us to distinguish between inhibitory mechanisms. Among the polyphenolic compounds tested for the inhibition of α-amylase α-glucosidase and AR so far were found to be competitive, noncompetitive and mixed inhibitors [4–10]. Such a variation in inhibitor potency, as well as the mode of enzyme inhibition, is not unusual since the inhibitor potency of polyphenolic compounds depends on several factors, such as the structure and stability of the inhibitors. As aforementioned XAD-7 methanolic extract composed of phenolic acid and anthocyanin, we tested the nature of the inhibition by F1 and F2 fractions of CO97216-1P/P. The initial velocity ‘v’ of the hydrolysis reactions catalyzed by α-glucosidase, pancreatic α- amylase, and aldose reductase were measured at various substrate concentrations [S] (1–5 mM) in presence and absence of F1 and F2 inhibitors[I] (10, 20, 30 and 50 μg/ml). We noted significant changes in the K_m_ and V_max_ in absence as well as the presence of these inhibitors. Double reciprocal plots for α-glucosidase and α-amylase respectively in the presence of extract inhibitors showed that straight lines were obtained with PNPG and CNPG3 substrates (Figs 7 and 8 and S1 Table). In the assay α-glucosidase had a K_m_ of 4.44 mM for PNP-glycoside and V_max_ value of 7.027 μmoles. In the presence of 10, 20, 30 and 50 μg/ml of phenolic acid compounds apparent V_max_ values were found to be 5.33, 4.18, 3.50 and 2.89 μmoles and K_m_ values changed to 7.29, 8.0 10.0, and 11.49 respectively (Fig 7A). In the presence of 10, 20, 30 and 50 μg/ml of anthocyanin compounds K_m_ values remained constant whereas V_max_ values changed to 3.05, 2.44, 1.88 and 1.32 μmoles respectively (Fig 7B). As 5-caffeoylquinic acid was found to be major phenolic acid, we tested the kinetics of α-glucosidase activity in its presence which showed changes in both Km and Vmax values (S1 Fig).

**Fig 9:**
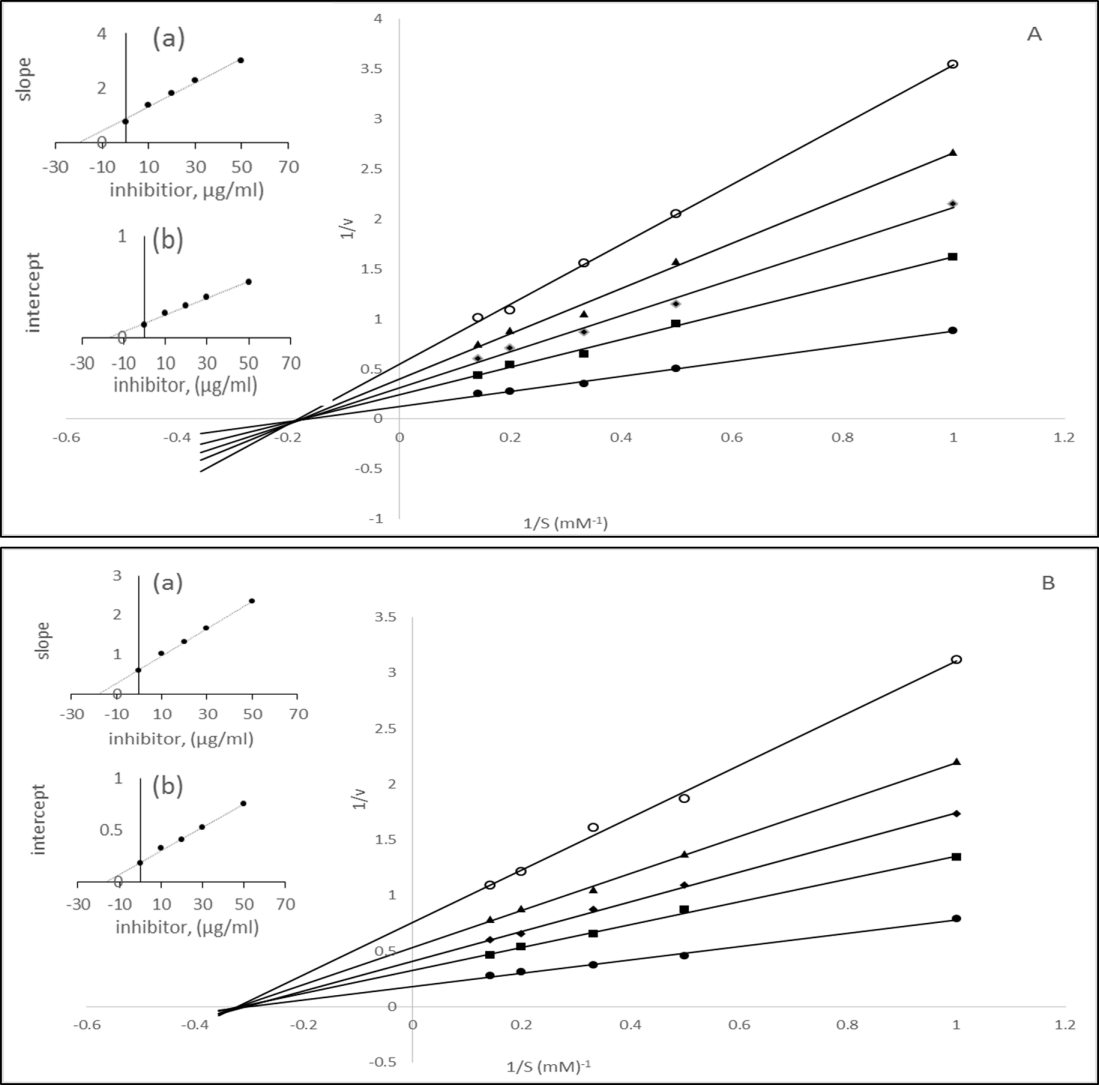
Lineweaver-Burk plot for the activities of aldose reductase in the presence of various concentration of substrates (1–5 mM) and inhibitors. A) different concentration of phenolic acids, (● 0, ▪ 10, ◊ 20 Δ 30, ○50 μg/ml) B) different concentration of anthocyanins (● 0, ▪ 10, ◊ 20 Δ 30, ○50 μg/ml).

In another assay for α-amylase K_m_ values increases and V_max_ values decreases with increase in the inhibitor concentration (Fig 8A and 8B). Thus in both assays for α-amylase and α-glucosidase we found that there was an increase in Km and decrease in V_max_ values with the increase in the concentration of phenolic acid fraction (F1). However, in the presence of anthocyanin fraction (F2) for α-glucosidase and α-amylase assay the K_m_ values remained constant and V_max_ values decrease. 5CQA also resulted in the increase in K_m_ and decreased in V_max_ values. Thus it is apparent that phenolic acid fraction (F1) behaved as mixed inhibitors for α-glucosidase and α-amylase whereas anthocyanin compounds acted as noncompetitive inhibitors. 5-caffeoylquinic acid acted as a mixed inhibitor of α-glucosidase and α-amylase. The results from aldose reductase assays in the presence of F1 and F2 are shown in Fig 9 (S1 Table). These results indicated that the binding of the phenolic compounds affected the velocity of the reaction catalyzed by aldose reductase proportionately to the concentration of the phenolic acid compounds in the reaction mixture, without affecting the Km (Fig 9A). In our assay, we found the values of K_m_ and V_max_ 3.03 mM and 9.70 μmoles/min respectively. The values of V_max_ changed to 6.13, 4.50, 3.70 and 3.14 in the presence of 10, 20, 30 and 50 μg/ml phenolic compounds respectively. The anthocyanin fraction F2 also showed a similar set of straight lines which meet at a single point in the concentration axis (Fig 9B). 5CQA resulted in the changes in the V_max_ values, but km remained constant (S1 Fig). Thus it was demonstrated that both phenolic and anthocyanin compounds acted as noncompetitive inhibitors of aldose reductase. The extent of changes in the K_m_ and V_max_ values under different fractions depends on the nature of the compounds. The affinity of the enzymes for the inhibitors varies and can be seen from the inhibition constants. The dissociation constant for competitive inhibition K_i_ was determined from the secondary plot of the slope of the straight lines of 1/v vs. 1/S against the inhibitor concentration. The intercept on the inhibitor axis of this plot gives the value of K_i_. The value of K_ii_, the inhibitor constant for noncompetitive inhibition, was obtained from a linear secondary plot of 1/V_max_ against the inhibitor concentration. The intercept on the inhibitor axis gives the K_ii_ values. The kinetic data for inhibition of α-amylase, α-glucosidase, and aldose reductase catalyzed hydrolysis reaction are shown in Table 5. For anthocyanin fraction F2, the value of K_i_ was equal to K_ii_ in the assays for α-amylase, α-glucosidase and aldose reductase, which are typical noncompetitive inhibitors. For phenolic compounds fraction F1, K_ii_ > K_i_ for α-glucosidase and α-amylase, as is the case of the mixed inhibitor with a major mode of inhibition being the competitive type. Similarly, chlorogenic acid acted mixed inhibitor against α-amylase and α-glucosidase. However, K_ii_ = K_i_ for aldose reductase as for noncompetitive inhibitors.

**Table 5:** Kinetic data on the inhibition of amylase, glucosidase and aldose reductase by phenolic and anthocyanin compounds.

enzyme	inhibitor	K_i_	K_ii_	K_ii_/K_i_	type of inhibition
α-amylase	F1	8.7	22.5	2.58	Mixed inhibition
	F2	17.5	17.2	0.98	Non-competitive
	5CQA	19.0	36.0	1.89	Mixed inhibition
α-glucosidase	F1	9.5	35.2	3.70	Mixed inhibition
	F2	18.5	18.2	0.98	Non-competitive
	5CQA	13.1	18.4	1.40	Mixed inhibition
aldose reductase	F1	12.6	12.1	1.04	Non-competitive
	F2	28.1	28.4	1.01	Non-competitive
	5CQA	10.1	10.3	1.01	Non-competitive

Some previous report on inhibitory potency of chlorogenic acid demonstrated that the binding position of caffeic acid to the quinic acid in chlorogenic acid has played an important role towards the inhibition of glucosidase activity. Xu et al. found that position of caffeoyl unit to the quinic acid affect the rate and mode of inhibition α-glucosidase with an order of 5CQA> 4CQA>3CQA [34]. In that study, they also found that dicaffeoylquinic acid was stronger than monocaffeoylquinic acid and acted as noncompetitive with a closure mixed type inhibition. From the kinetic data in our assay, we observed that the phenolic acid group containing caffeoylquinic acids and its isomers acted as mixed type inhibitors against α-glucosidase. In a separate study of 5-caffeoylquinc acid, it was seen to be a mixed inhibitor (S1 Fig) of α-glucosidase. Several anthocyanin extracts have been reported as competitive, mixed and noncompetitive inhibitors α-glucosidase. You et al. found that cyanidin derivative of Musscadine acted as noncompetitive inhibition of glucosidase [33]. Pelargonidin derivatives acted as a noncompetitive inhibitor against α-glucosidase. The anthocyanin extract of purple fleshed potato cultivar CO97216-1P/P containing petunidin, cyanidin, and malvidin derivatives showed a noncompetitive inhibition in our study. Narita et al. reported that chlorogenic acid acted as a mixed inhibitor in a kinetic study of inhibition of pancreatic α-amylase [35]. Similar to α-glucosidase the structural difference in caffeoylquinic acid also influences the inhibitory activity of amylase. 5CQA had higher inhibition activity compared to 4CQA and 3CQA. A detail kinetic of inhibition by young apple polyphenols (YAP) containing chlorogenic acid showed mixed type inhibition of α-amylase [36]. In a similar study the methanolic extract of finger millet, which consists of various phenolic compounds, such as gallic acid, caffeic acid, ferulic acid, showed strong inhibition towards glucosidase and pancreatic amylase with a noncompetitive inhibition [29]. In a similar way, the phenolic acids containing caffeoylquinic acid acted as a mixed inhibitor of α-amylase in our assay. Various anthocyanin compounds have been reported as effective inhibitors of α-amylase. Delphinidin, cyanidin, Malvidin, peonidin aglycon derivatives from cherries showed competitive inhibition against α-amylase [37]. The anthocyanins present in the CO97216-1P/P showed a noncompetitive inhibition. Several reports are available on aldose reductase inhibition by natural products from medicinal plants, food, and vegetables [38]. The major group of compounds comprised phenolic compounds such as flavonoids, phenolic acids, anthocyanins and other bioactive compounds. In a study of inhibition behavior of some phenolic acids (tannic acid, chlorogenic acid, and p-coumaric acid) reported that chlorogenic acid and other phenolic acid acted as mixed and noncompetitive inhibitors of rat kidney aldose reductase [39]. Chlorogenic acid extract from *Wrightia tinctoria* was found to be an uncompetitive inhibitor of aldose reductase [40]. Finger millet phenolic acid compounds consisting gallic acid, p-coumaric acid, ferulic acid showed a noncompetitive inhibition towards aldose reductase [41]. Yoshimoto et al. reported that methanolic extract of sugarcane composed of quinic acid, caffeic acid and its derivative chlorogenic acid is a potent inhibitor of aldose reductase with IC_50_ values 0.14 mg/ml [42]. Yawadio et al. reported that black and brown rice with anthocyanin compounds cyandin -3-glucoside, peonidin-3-glucoside, ferulic acid, and tocopherol are found to be a potent inhibitor of aldose reductase with an IC_50_ value of 8.7 to 27.5 μg/ml against human recombinant AR [43]. Although various anthocyanin extracts have been reported as effective inhibitors of aldose reductase data on inhibition kinetics are scanty. The anthocyanin compounds from purple fleshed potato tubers CO97216-1P/P showed a noncompetitive inhibition towards aldose reductase.

## Conclusions

Colorado State University postharvest program have been screening the potato cultivars and advanced selections for improved nutrition and health benefits. This study indicated that colored flesh potato tubers contain a mixture of several phenolic compounds ranging from phenolic acid to anthocyanins. We have separated, isolated and characterized the group of phenolic acid and anthocyanin compounds in colored flesh potatoes. It is interesting to note that these group of compounds had significant inhibitory activities on α-glucosidase, α-amylase, and aldose reductase. These phenolic compounds acted effective non-competitive inhibitors and mixed with carbohydrate-hydrolyzing enzymes such as α-amylase and α-glucosidase. Thus polyphenol rich potato tubers have the potential to interfere with or delay absorption of dietary carbohydrates in the small intestine, leading to suppression of postprandial blood glucose sugars. However, there are reports of a problem on the direct intestinal absorption of anthocyanins and phenolic compounds. It was mentioned that about 1% or less of ingested anthocyanin is estimated to be incorporated into plasma. There is a possibility of biotransformation of these phenolic compounds after consumption. Food-grade phenolic inhibitors from potatoes are potentially safer, and therefore may be preferred alternatives for inhibition of carbohydrate breakdown and control of glycemic index. Additionally, the significant inhibitory effect on aldose reductase suggests that phenolic fractions in potato tubers can possess constituents with antidiabetic and inhibitory effects on diabetic complications.

## Supporting information

S1 Fig Lineweaver-Burk plot for the activities of α-glucosidase (A), α-amylase (B), and aldose reductase (C) in the presence of various concentration of substrates (1–5 mM) and inhibitors. different concentration of 5-caffeoylquinic acid, (● 0, ▪ 10, ◊ 20 Δ 30, μg/ml), and a) and b) are the secondary plots for K_i_ and K_ii._
(TIF)

S1 Table The values used to make the Table 4 and Figs 3, 7, 8 and 9. (XLSX)
