# Supplementary figures and images for: Inhibition of α-glucosidase, α-amylase, and aldose reductase by potato polyphenolic compounds
Source: PLoS One. 2018 Jan 25;13(1):e0191025. doi: 10.1371/journal.pone.0191025 (PMC5784920; doi:10.1371/journal.pone.0191025)

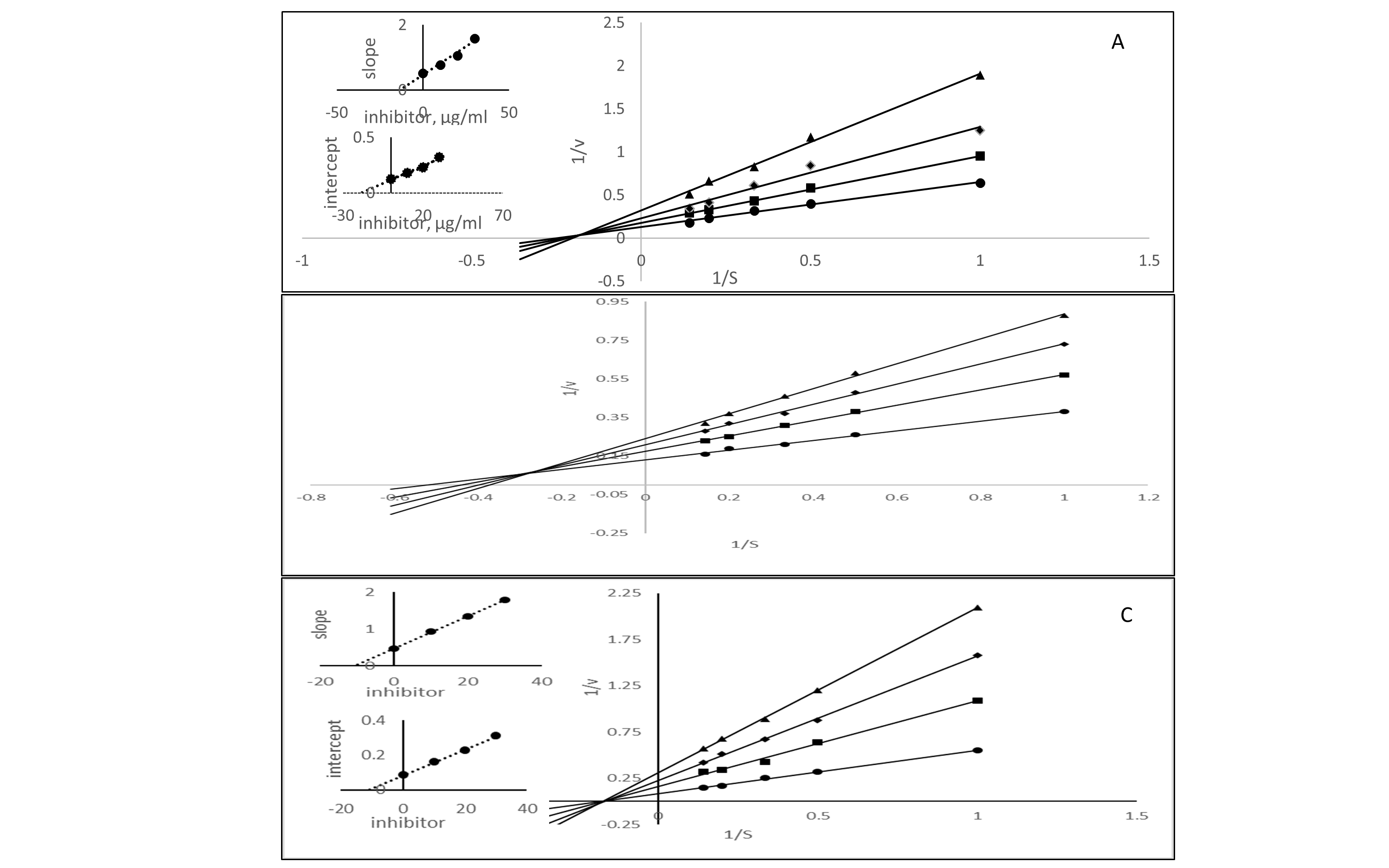

Supplement: S1 Fig — Lineweaver-Burk plot for the activities of α-glucosidase (A), α-amylase (B), and aldose reductase (C) in the presence of various concentration of substrates (1–5 mM) and inhibitors. different concentration of 5-caffeoylquinic acid, (● 0, ▪ 10, ◊ 20 Δ 30, μg/ml), and a) and b) are the secondary plots for Ki and Kii. (TIF) [file pone.0191025.s001.tif]
